# Saliva based diagnostic methodologies for a fast track detection of autism spectrum disorder: A mini-review

**DOI:** 10.3389/fnins.2022.893251

**Published:** 2023-01-04

**Authors:** Vaibhav Sharma, Saptamita Paul Choudhury, Saroj Kumar, Fredrik Nikolajeff

**Affiliations:** ^1^Department of Health, Education and Technology, Luleå University of Technology, Luleå, Sweden; ^2^School of Biotechnology, Kalinga Institute of Industrial Technology, Bhubaneswar, Odisha, India; ^3^Department of Biophysics, All India Institute of Medical Sciences, New Delhi, India

**Keywords:** autism disorder, biomarkers, diagnosis, saliva, proteomic and RNA biomarkers

## Abstract

Autism spectrum disorder (ASD) is considered a complicated neurodevelopment disorder with rising prevalence globally. ASD is characterized by a series of events including varying degrees of defects in communication, learning, and social interaction which is accompanied by stereotypical behavioral patterns. Despite extensive research, the current diagnosis for ASD is complex and almost solely based on the behavioral assessments of the suspected individuals. The multifactorial etiopathology of this disease along with the diversity of symptoms among different individuals adds to the current intricacies for accurate prognosis of ASD. Hence, there exists a dire need for biologically relevant biomarkers for an early diagnosis and for tracking the efficacy of therapeutic interventions. Until recently, among various biofluids, saliva has gained increasing interest for biomarker identification, the advantages include the non-invasive nature and ease of sample handling. This mini-review aims to provide a succinct summary of recent literature on saliva-based diagnostic modalities for ASD, examine various studies that highlight the potential use of proteomic and/or RNA-based biomarkers. Finally, some conclusive perspectives of using the salivary system for ASD mechanistic details and diagnosis are also discussed.

## 1. Introduction

**FIGURE 1:**
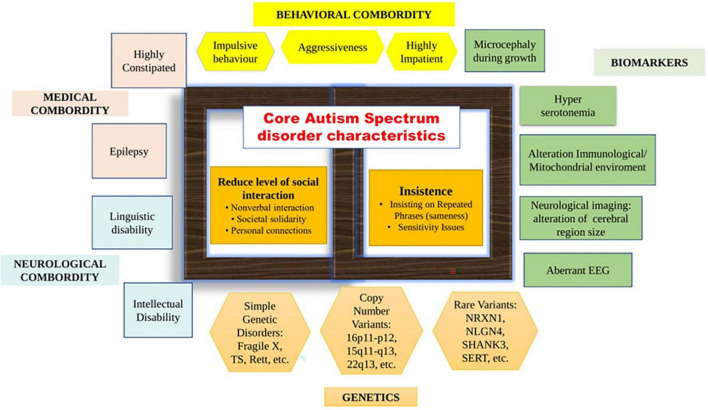
Autism spectrum disorder (ASD) characteristics, complications, and biomarkers. In the middle of the figure, the key characteristics of ASD are depicted, as well as the typical traits necessary for a diagnosis. As per the Diagnostic and Statistical Manual of Mental Disorders-V (DSM-V) draught standards, all three social connectivity/interactions symptoms must be present to earn a diagnosis. One sector shows the deficiency of proper socialization and networking, which is one of the “negative characteristics” of ASD. In the DSM-V draught criteria, restricted/recurrent behavior signs are necessary for a diagnosis. Another sector indicates the occurrence of unusual limited, repetitive, or sensory activities, which are considered “positive signs” of ASD. Symptoms or biomarkers that are not necessary for an ASD diagnosis but are more frequent in ASD than in the regular populace can be found throughout the perimeter of the picture. A large range of complicated disorders or signs, including neurological, behavioral/psychiatric, and medical areas, are observed in a significant minority or perhaps a majority of people with ASD. Biomarkers and genetic data demonstrate high variation among individuals with ASD, as predicted given the wide range of comorbidity symptoms.

Autism spectrum disorder (ASD), generally referred to as autism, is a widespread, genetically determined, and diverse neurological illness having fundamental mental abnormalities along with autism’s habits, abilities, and problems (Lord et al., 2020). The intended inclusion of the spectrum phrase in 5th version of the Diagnostic and Statistical Manual of Mental Disorders (DSM-V) diagnosis of “ASD” represents a highly multifaceted and diverse condition noteworthy for its wide range of symptoms and as far as its essential characteristics are concerned, there is a great deal of variation (Figure 1) in all spheres. ASD seems to have a greater prevalence in boys than girls, according to epidemiologic administration as well as society surveys, recorded values range around 2:1–5:1, with a 4:1 estimate in the 2010 Global Burden of Disease study (Loomes et al., 2017). Probably of the utmost difficult sections of ASD is its etiology. The hypothesized explanations of ASD varied all in the overall spectral region and are also thought to include the children’s intrauterine environments, early developmental childhood, and heritability. According to recent data, hereditary, ecological, proinflammatory, immunologic, as well as metabolism variables all have a part in the condition (Zhang et al., 2020). Diagnosis is carried out under as per the guidelines in DSM-V (Janšáková et al., 2021). Suspects are also diagnosed using the Autism Diagnostic Observation Schedule 2nd version (ADOS-2) and the Autism Diagnostic Interview-Revised (ADI-R) (Lord et al., 1994; Lord et al., 2000). Each of the aforementioned diagnostics is globally regarded as the gold standard and is attributed to the fact of behavioral signs within individuals as well as a conversation with the associated patient’s health care professional (Janšáková et al., 2021).

Biomarkers represent premonitory signals that allow either early identification or the determination of the desired result during a relatively primordial phase of illness. The scientific evidence for the diagnosis comes from blood, urine, and cerebral spinal fluid. Biomarkers are employed as an indication of a physiological element that indicates a preclinical symptom, degree of the ailment, or counterfeit symptom of the illness in certain situations (Mayeux, 2004). One such category of biological indicators could be used for a variety of purposes, including- (1) screening those who are prone to be impacted or who have been among the “preclinical” phases of the disease, (2) reducing illness variability in medical research or etiological research, (3) reflecting the genetic background of illness, including initiation, response time, and diagnosis, and (4) serving as a diagnostic testing goal (Mayeux, 2004). Biological markers for a variety of neurological illnesses have been identified due to recent advancements. The phosphorylated tau protein and aggregated β-amyloid peptide for Alzheimer’s disease (AD), α-synuclein-containing Lewy bodies and altered dopamine transporter (DAT) imaging for Parkinson’s disease (PD) (Choudhury et al., 2022), superoxide dismutases (SOD) mutations for familial amyotrophic lateral sclerosis (ALS), and CAG repeats caused by Huntington’s gene mutations in Huntington’s disease are only a handful of several novel biological markers lately discovered (Rachakonda et al., 2004).

The pathogenesis of ASD is multifaceted, with genetic, environmental, parent aging, and medical issue all playing a role. Demonstrating a validated marker biomarker is difficult because the etiology of ASD is still mostly unclear. Researchers around the globe are seeking these indicators mostly in the bloodstream, amniotic fluid, or saliva (Baron-Cohen et al., 2015; Pivovarciova et al., 2016). Saliva is an underappreciated bodily element with several benefits and capacities as salivary sampling is non-invasive, simple, and can be conducted several times within a short amount of time, therefore skilled personnel is not required. Screening of salivary indicators might be used not only for medical diagnostics but also to track the progression of illnesses (Roi et al., 2019). Therefore, this review is aimed to summarize the recent literature on saliva-based diagnostic modalities about ASD, examine various studies that highlight the potential use of proteomic and/or RNA-based biomarkers.

## 2. Saliva: An alternate diagnostic biofluid for ASD

The search for biomarkers, particularly those useful in the diagnosis of neurological diseases, is an extremely difficult undertaking. In scientific and medical diagnosis, the use of plasma or amniotic fluid is still regarded as the benchmark (Janšáková et al., 2021). On average, saliva is being utilized to assess oral and systemic illnesses, however in the case of other illnesses, the oral fluid is subordinate to some other stated bodily secretions or tissue (Janšáková et al., 2021). ASD patients are a distinct set of population, and saliva collections in ASD children may be more challenging as compared to other neurotypical children. Some ASD patients might well have lower saliva production, dryness mouths, or subjectively documented xerostomia (Blomqvist et al., 2015). Elevated whole-blood 5-HT, or hyper-serotonemia, was the initial biomarker found in autism 50 years ago (Aaron et al., 2019), and it was distinct to autism amongst all other neurological illnesses. However, collecting saliva is lesser distressing and non-invasive than collecting blood, therefore it appears that ASD youngsters will comply more easily. The two fundamental methods for collecting saliva are the extraction of unstimulated whole mouth saliva (UWMS) which begins with passively dripping of salivary fluid inside the oral cavity, followed by the discharge of the full oral contents into the clean tubes. Secondly, different stimulants, including cotton swabs, citric acid, chewed paraffin blocks, or filtering papers, are used to enhance saliva secretion (Granger et al., 2007). Although UWMS might well be challenging to collect from such patients in certain situations, research has shown that ASD patients can provide a greater quantity of saliva than standards (Janšáková et al., 2021). Biochemical variation and/or repeatability may play a major influence in saliva-based autism investigation for a variety of explanations. However, autism has been linked when there is a slightly reduced level of dental hygiene (Zhang et al., 2020). Several investigations, on the other hand, have found that the prevalence of decaying, missing, repaired, as well as permanent teeth in the main dentition, is less in infants with ASD than in healthier subjects (Kuter and Uzel, 2021). Furthermore, it appears that the prevalence of cavities in the ASD community varies by nation (Zhang et al., 2020). Dietary interests and accompanying feeding behavior, like selection, rejects, and intolerances are seen in ASD (Malhi et al., 2017; Leiva-García et al., 2019), and a high inclination for nutritionally poor diets (Ahearn et al., 2001), could be another factor impacting saliva variation. Dietary habits have an impact on both the total intestinal flora and the oral microbiota (Bolte, 1998; Finegold et al., 2002), which have been reported to vary in people with ASD (Hicks et al., 2018b). Additional changes in biomarkers from saliva are generated *via* biases inside the composition of the bacterial cavity induced by favoring certain foods whilst avoiding others (Malhi et al., 2017; Leiva-García et al., 2019). Sometimes the proportion of some indicators in the saliva may be altered by different collection strategies and methods which are utilized to stimulate salivary production, an example of this could be differences in salivary cortisol obtained from stimulated vs. unstimulated method of saliva collection (Holm-Hansen et al., 2004; Putnam et al., 2012; Nunes et al., 2015). Selecting the perfect salivary extraction methodology might be much more challenging than it appears. The usage of, for example, citric acid for salivary activation with a person might not even suit, others might prefer, for example, stimulating *via* paraffin chew, or vice versa. Approval of a collecting approach is indeed dependent on ASD symptoms, although it does not appear to be an issue in general (Putnam et al., 2012).

Saliva includes biological and oral microbiological DNA and RNA that can be used as biological markers for the prognostication and early detection of a range of illnesses, along with ASD, but there are two major drawbacks: many of the secluded DNA and RNA comes from the verbal microbial communities, and indeed the quantity of isolated human nucleic acids is minimal (Ostheim et al., 2020). As a result, appropriate saliva sample collection, and treatment procedures must be developed following the results of the study. Numerous non-overlapping genes were discovered in massive genome-wide association studies aimed at detecting connections among genetic variations and illness, emphasizing the extensive genetic variability of ASD (Bralten et al., 2018). The Simons Foundation Autism Research Initiative (SFARI) Gene Database now contains structured data with all identified human genomes linked to ASD. Several of those are required for neural improvement, operate as transcriptional regulators, or service apart from chromosome remodeling (Bralten et al., 2018). Considering the prospect of saliva as a source of biological material for genetic and transcriptome study, ASD investigation has primarily relied on nucleic acids extracted *via* blood. Only few studies concerning salivary nucleic acid research has been published to date. Utilizing saliva samples from ASD patients, Braam et al. (2013) investigated six single nucleotide polymorphisms (SNPs) in CYP1A2. Researchers discovered that the loss of exogenous melatonin effectiveness could be due to a delayed melatonin metabolism induced by an SNP within the CYP1A2 gene, which could also be linked to the processes which induce autism. Whole-exome and whole-genome sequencing have been frequently employed in genomic investigations across huge ASD populations since the emergence of newer techniques. Feliciano et al. (2019) verified the feasibility of employing salivary samples for whole-exome sequence analysis among individuals with ASD. In 10.4% of individuals despite previous genomic discoveries, they discovered variations in alleles and locations which are medically recognized origins or substantial contributions to ASD, as well as variations that are probably related to ASD in another 3.4% of families (Feliciano et al., 2019). The study discovered 34 genes with harmful variations, 21 of which have been linked to ASD or neurological diseases. BRSK2 has been proposed as an increased ASD danger allele, bolstering the evidence for the involvement of other genes (FEZF2, ITSN1, PAX5, DMWD, and CPZ) in ASD (Feliciano et al., 2019). These observations are also in line with earlier research that supports the female protective model (which explains why men have a greater frequency of ASD than women) (Jacquemont et al., 2014). Research involving groups of thousands of people of both genders and their descendants will be required for additional elucidation of the genomic danger variables related to ASD, comprehension of the genetic etiology of ASD, and the likely genetic origin of the higher incidence in men. Though, it is also important to mention some limitations of saliva-based analysis also, the detection of large molecular weight or charged biomolecules is not possible with saliva, as these do not get from plasma to saliva. Bias may come from eating, drinking, chewing gum, or smoking (Celec et al., 2016). Finally, the analyte concentration might be less in saliva when compared with blood plasma, but this can be easily circumvented using highly sensitive analytical technique or a higher sample volume. In the foreseeable future, non-invasive salivary collection, as well as the prospect of getting high-quality genetic data through salivary DNA analysis, might assist and encourage research to attract more individuals.

## 3. Proteomic and RNA based biomarkers in ASD

Proteomics is a strong technique for studying biological mechanisms, and it can help researchers find biological markers for medical diagnosing, therapy management, and pathophysiology pathways in autism (Abraham et al., 2019). In contrary to the several other scientific methodologies utilized in autism research, a few proteomic-based investigations have been conducted to date (Abraham et al., 2019). Twelve investigations of serum or plasma, one examination of periphery B lymphocytes, three analyses of salivary, and one investigation of urinary were used in independent or worldwide proteome analyses of ASD (Abraham et al., 2019). Lately, this has gained a lot of focus on finding biomarkers that could help with earlier detection, prediction, and therapy responsiveness (Zhang et al., 2020). A proteomic mass spectrometric investigation may result in the finding of biological markers within individual bodily fluid such as saliva. Saliva proteome of person without ASD was studied utilizing mass spectrometry (MS) technology as well as contrasted to neurotypical controls people in such an effort to improve its investigation toward a salivary proteomics biomarker (Ngounou Wetie et al., 2015b). Numerous salivary molecules notably elevated prolactin-induced protein, lactotransferrin, Ig kappa chain C, Ig chain 1 region C, Ig lambda–2 chains C, neutrophil elastase, and polymeric immunoglobulin receptor, revealed significantly substantial changes (Zhang et al., 2020). Proteins involved in immunological responses are likely to be unregulated since the immunological responses are thought to have an involvement in ASD pathogenesis (Farah et al., 2018). Studies on ASD-related neuroimmune abnormalities, as well as the link between ASD and parental autoimmune illnesses, support the idea that immune system dysregulation is either etiological or catastrophic to ASD (Farah et al., 2018). Ngounou Wetie et al. (2015b) undertook a pilot investigation to look for differences for salivary protein levels and characteristics amongst ASD sufferers and normal individuals in the hopes of finding novel ASD salivary biomarkers. Researchers were able to identify diversity in saliva proteome levels for ASD patients using MS-based proteomics, with 12 proteins possessing an enhanced level and 4 proteins exhibiting a lower content in autism patients’ saliva when matched to normal subjects (Ngounou Wetie et al., 2015b). The following table (Table 1) summarizes the comparative picture of biomarkers pertaining to ASD within the saliva and blood plasma of ASD patients, alongside their biological activities. Most of the proteins described are implicated in immunological responses. Following up on the protein-protein interconnections, it was discovered that lactotransferrin (LTF) and prolactin Inducible protein (PIP) are also involved in interaction with the hormone prolactin (Farah et al., 2018). Submaxillary gland androgen-regulated protein 3B, statherin, histatin, and other proteins in the submaxillary gland were discovered in the same survey where they all interact with others (Farah et al., 2018). Although many proteins were confined in either saliva or blood plasma. Some molecules like oxytocin, cortisol, and testosterone were found in both saliva and plasma of ASD patients (Bakker-Huvenaars et al., 2020; Pinto et al., 2021) (Table 1).

**TABLE 1:** Summarizes a comparative picture of biomarkers within the saliva fluid and blood plasma of Autism spectrum disorder (ASD) patients, along with their biological activities.

Biomarker	Function	Protein length	Frequency	Biofluid	References
Lactoferrin/Lactotransferrin (LTF)	Innate immunity, antibacterial activity, antifungal activity	711 A.A	Higher	Saliva	Beljaars et al., 2004
Prolactin inducible protein (PIP)	Binding of IgG antibody, binding of actin, aspartic-type endopeptidase activity, regulation of T-cell apoptosis, regulation of immune system	146 A.A	Higher	Saliva	Hassan et al., 2008
Ig kappa chain C region (IGKC)	Constant regions of immunoglobulin heavy chains	107 A.A	Higher	Saliva	Farah et al., 2018
IgG gamma-1 chain C region (IGHG1)	Humoral immune response	330 A.A	Higher	Saliva	Farah et al., 2018
Annexin A1 (ANXA1)	Anti-inflammatory activity, regulation of immune system, wound repair, chemotaxis, neutrophil extravasation	346 A.A	Higher	Saliva	Ernst et al., 2004; Blume et al., 2012; Weyd et al., 2013
Neutrophil-defensin 1 (DEFA1/DEFA1B)	Chemotaxis, antimicrobial immune response, Gram-positive antibacterial, innate immune response	94 A.A	Higher	Saliva	Szyk et al., 2006; Yamaguchi and Ouchi, 2012
Neutrophil elastase (ELANE)	Calcium homeostasis, negative regulation of chemotaxis and inflammation, Gram-negative antibacterial, down-regulation of chemokine production, up-regulation of IL-8 production, and MAP kinase activity, transcriptional repressor	267 A.A	Higher	Saliva	Papareddy et al., 2010
Salivary acidic proline-rich phosphoprotein (PRH1/2)	Calcium-binding, inhibition of calcium phosphate crystal formation, inhibition of calcium carbonate precipitation, maintenance of oral health	166 A.A	Lower	Saliva	Bennick et al., 1981
Statherin (STATH)	Inhibition of calcium phosphate precipitation, and crystal growth, enamel boundary lubricant, oral bacteria colonization	62 A.A	Lower	Saliva	Raj et al., 1992
Histatin-1 (HTN1)	Inhibition of calcium phosphate precipitation, and crystal growth, antifungal, wound healing, enamel pellicle formation	57 A.A	Lower	Saliva	Farah et al., 2018
C4b-binding protein	Role in inflammation and regulate compliment activation	587 A.A	Lower	Plasma	Warren et al., 1994
Oxytocin	Role in human behavior and social interactions	9 A.A	Lower	Plasma and saliva	Modahl et al., 1998; Bakker-Huvenaars et al., 2020
Testosterone	Regulates sexual drive, bone mass and fat distribution	–	Lower	Plasma and saliva	Croonenberghs et al., 2010
Gamma-aminobutyric acid (GABA)	Primary inhibitory neurotransmitter for the central nervous system (CNS)	–	Higher	Plasma	Dhossche et al., 2002

Further investigation performed by a similar investigative group found that peptides associated with metabolic distress and lipids metabolisms, such as apolipoproteins A1 and A4 and Zn alpha2 glycoprotein, have been likewise altered among ASD specimens when contrasted to normal adolescents (Ngounou Wetie et al., 2015a). Such observations were aligned to the idea of a modification in resistance, oxidative stress, and lipids metabolic in individuals with ASD, that may be detected in saliva specimens using the biomarkers stated (Scott and Dhillon, 2007; Zhang et al., 2020).

MicroRNAs (miRNAs) are single-stranded RNA sequences that interact with the expression of genes by binding to target mRNA and controlling protein translation. In ASD, a variety of miRNA expression patterns have been discovered, the majority of which are associated with central nervous formation and functioning (Abraham et al., 2019). A few of the detected miRNAs are found within the human cerebral, where they regulate many biological activities such as prenatal and adult neurogenesis, brain development, and synaptic plasticity (Presumey et al., 2017). One of the main prevalent research disadvantages is the discovery that most of the down-regulated miRNAs are not unique to ASD and yet are seen in different neurological diseases (Mead and Ashwood, 2015). The wide range of down-regulated miRNAs seen in ASD patients is most likely due to the several pathways implicated in the disease’s genesis. Furthermore, various other symptoms which are not regarded as fundamental characteristics but impact a significant fraction of people with ASD, such as intestinal abnormalities or immunological diseases, are prevalent in ASD (Abraham et al., 2019).

In earlier investigations, differential expression patterns of miRNAs in patients with ASD have been discovered in research, suggesting that miRNAs might be used as biomarkers for ASD screenings. Brain tissue, blood, and cultured peripheral lymphoblast were utilized to identify miRNA in ASD patients (Ngounou Wetie et al., 2015b). However, saliva, on the other hand, appears to be a handy biomaterial for finding miRNAs biomarkers, and a favored sample for families of ASD sufferers, as per latest investigations. During later times, Hicks et al. (2016, 2018a) investigated the efficacy of saliva miRNA as ASD indicators with details. Researchers looked at the possibility of saliva miRNAs as diagnosing detection methods for ASD in pilot research published in 2016 (Hicks et al., 2016). Using short RNA sequencing, they discovered 14 miRNAs that were generated differentially among ASD individuals compared to neurotypical youngsters (Hicks et al., 2016). Four from these 14 miRNAs were shown to be deregulated in saliva samples from ASD patients (miR-23a-3p, miR-27a-3p, miR-30e-5p, and miR-32-5p), whereas the rest 10 were found to be increased (miR-140-3p, miR-2467-5p, miR-218-5p, miR-335-3p, miR-628-5p, miR-7-5p, miR-191-5p, miR-127-3p, and miR-3529-3p) (Farah et al., 2018). The 78 salivary RNAs earlier discovered in juvenile ASD were evaluated in the latest long-term investigation to discover whether these remained disrupted in youngsters and/or changed with medical approaches (Levitskiy et al., 2021). Seven RNAs (four miRNAs, two piRNAs, and one microbial RNA) was linked together with the scores on the Vineland Adaptive Behavioral Scales 2nd Edition (VABS-II), the Autism Spectrum Quotient (AQ), and the Behavioral Assessment System for Children (BASC) among the old population (Zhang et al., 2020). Twelve saliva RNAs have been discovered as altering through times among individuals with ASD (Zhang et al., 2020). Furthermore parallel, within the aging population, three miRNAs appeared to be linked to behavioral characteristics which altered across times within the junior group. Such miRNAs play an important role in networks linked to cerebral formation as well as performance. A study of the discovered miRNAs, on the other hand, revealed that just a few short RNAs remain constant throughout investigations. MiR-146b-5p, piR-6463, piR-24085, and miR-148a-5p, for example (Levitskiy et al., 2021), are linked with behavior features and either vary with times during long-term research and then seem to have been incorporated into the diagnosis panels (Hicks et al., 2018a) or detected by RNA sequencing (Hicks et al., 2020). RNA sequencing and qPCR were also used to identify miR-23a-3p as being overexpressed. Such observations give early indications that RNAs can be used to track behavioral patterns, therapy efficacy, and prognosis among people with ASD (Zhang et al., 2020). The above results give indications that RNAs can be used to track development progression, therapy efficacy, and prognosis in people with ASD. The researchers found five differently expressed miRNAs and a distinct abundance of a particular bacteria in ASD relative to standards using a combination of miRNAs expression profiling and 16S RNA microbiome analysis of saliva from ASD and neurologically unaffected controls (Janšáková et al., 2021). The unfavorable link between saliva miR-141-3p production and *Tannerella* quantity was the most important of the identified miRNA/bacteria correlations (Janšáková et al., 2021). The presence of miRNAs and microbiome dysregulation in the saliva of ASD youngsters may be linked to the sufferers’ mental issues (Janšáková et al., 2021). These findings imply that ASD symptoms may cause changes in miRNAs expression and the microbiota (Janšáková et al., 2021). As a result, combining the analysis of various types of RNA expression with microbial community analysis, as well as genome testing, appears to be the greatest favorable procedure of acquiring effective outcomes for a greater knowledge of ASD etiopathology and the possible future use of these compounds for the diagnostics and therapeutics of ASD, as well as lessening of its medical symptoms.

## 4. Concluding remarks and future perspectives

The benefits of salivary fluid for autism investigation as well as its possible therapeutic use cannot be overstated. Numerous elements must be addressed, or a plan for saliva studies and salivary utilization should be devised. Generally, the technological and operational characteristics of the studied marker should be considered. To limit the possible diversity in the realm of neurodevelopmental disorders, particularly ASD, homogeneous sets outlining specific categories, solely focusing on age group and intestinal and dietary history, would be proposed. The use of a salivary system inside the diagnosing procedure is contingent on the discovery of potential ASD biomarkers. The finding of these biomarkers, nevertheless, was not the sole purpose of the salivary study; it also necessitates broad investigation on several biological materials. Thereafter, saliva can be used as a non-invasive additional method with all of its benefits. The recent discovery of efficient isolation protocols from salivary exosomes has aid in the hope of an improved and timely diagnosis of neurodegenerative diseases (Rani et al., 2019; Rani et al., 2021; Rastogi et al., 2021), similar approaches may be tried for ASD diagnosis as well. Given some of the aforementioned considerations, the identification of biomarkers from multiple perspectives will aid in improving knowledge of ASD and disclose the pathophysiology of all such illnesses.

## Author contributions

VS and FN conceptualized the work. VS and SC wrote the manuscript. SK, VS, and FN reviewed the manuscript. All authors contributed to the article and approved the submitted version.
